# Supplementary material for: HIVprotI: an integrated web based platform for prediction and design of HIV proteins inhibitors
Source: J Cheminform. 2018 Mar 9;10:12. doi: 10.1186/s13321-018-0266-y (PMC5845081; doi:10.1186/s13321-018-0266-y)
Supplement: Supplementary file 2 — Additional file 2. Source code of HIVProtI web server. [file 13321_2018_266_MOESM2_ESM.zip › HIVprotI_Source-code/hivprotI/weka-3-6-6/doc/weka/gui/sql/DbUtils.html]

DbUtils


---


|  |  |  |
| --- | --- | --- |
| **Overview** \| **Package** \| **Class** \| **Tree** \| **Deprecated** \| **Index** \| **Help** \| **Weka's home** | |  |
| **PREV CLASS**   **NEXT CLASS** | **FRAMES**    **NO FRAMES**     **All Classes** |  |
| SUMMARY: NESTED \| FIELD \| CONSTR \| METHOD | DETAIL: FIELD \| CONSTR \| METHOD |  |


---


## weka.gui.sql Class DbUtils

```
java.lang.Object
  weka.experiment.DatabaseUtils
      weka.gui.sql.DbUtils
```

**All Implemented Interfaces:**: java.io.Serializable, RevisionHandler

---

``` public class DbUtils extends DatabaseUtils ```

A little bit extended DatabaseUtils class.

**Version:**
:   $Revision: 1.4 $

**Author:**
:   FracPete (fracpete at waikato dot ac dot nz)

**See Also:**: `DatabaseUtils`, `DatabaseUtils.execute(String)`, Serialized Form

---

**Field Summary**

**Fields inherited from class weka.experiment.DatabaseUtils**

`BOOL, BYTE, DATE, DOUBLE, EXP_INDEX_TABLE, EXP_RESULT_COL, EXP_RESULT_PREFIX, EXP_SETUP_COL, EXP_TYPE_COL, FLOAT, INTEGER, LONG, PROPERTY_FILE, SHORT, STRING, TEXT, TIME`


**Constructor Summary**

`DbUtils()`
  
          initializes the object.


| **Method Summary** | |
| --- | --- |
| `java.sql.Connection` | `getConnection()`             returns the current database connection. |
| `java.lang.String` | `getRevision()`             Returns the revision string. |

**Methods inherited from class weka.experiment.DatabaseUtils**

`arrayToString, close, close, connectToDatabase, createExperimentIndex, createExperimentIndexEntry, createResultsTable, databaseURLTipText, debugTipText, disconnectFromDatabase, execute, experimentIndexExists, getDatabaseURL, getDebug, getKeywords, getKeywordsMaskChar, getPassword, getResultFromTable, getResultSet, getResultsTableName, getSupportedCursorScrollType, getUsername, isConnected, isCursorScrollable, isCursorScrollSensitive, isKeyword, maskKeyword, passwordTipText, processKeyString, putResultInTable, select, setDatabaseURL, setDebug, setKeywords, setKeywordsMaskChar, setPassword, setUsername, tableExists, translateDBColumnType, typeName, update, usernameTipText`

**Methods inherited from class java.lang.Object**

`equals, getClass, hashCode, notify, notifyAll, toString, wait, wait, wait`

**Constructor Detail**

### DbUtils

```
public DbUtils()
        throws java.lang.Exception
```

:   initializes the object.

    **Throws:**: `java.lang.Exception` - in case something goes wrong in the init of the DatabaseUtils constructor **See Also:**: `DatabaseUtils`


**Method Detail**

### getConnection

```
public java.sql.Connection getConnection()
```

:   returns the current database connection.

    :   **Returns:**: the current connection instance

---


### getRevision

```
public java.lang.String getRevision()
```

:   Returns the revision string.

    :   **Specified by:**: `getRevision` in interface `RevisionHandler` **Overrides:**: `getRevision` in class `DatabaseUtils`
    :   **Returns:**: the revision


---


|  |  |  |
| --- | --- | --- |
| **Overview** \| **Package** \| **Class** \| **Tree** \| **Deprecated** \| **Index** \| **Help** \| **Weka's home** | |  |
| **PREV CLASS**   **NEXT CLASS** | **FRAMES**    **NO FRAMES**     **All Classes** |  |
| SUMMARY: NESTED \| FIELD \| CONSTR \| METHOD | DETAIL: FIELD \| CONSTR \| METHOD |  |


---
